# The First African Swine Fever Viruses Detected in Wild Boar in Hong Kong, 2021–2023

**DOI:** 10.3390/v17070896

**Published:** 2025-06-25

**Authors:** Karina W. S. Tam, Candy C. Y. Lau, Timothy T. L. Ng, Sin Ming Ip, Sin Fat Pun, Amanda Corla, Carrie Batten, Christopher J. Brackman

**Affiliations:** 1 Agriculture, Fisheries and Conservation Department, The Government of the Hong Kong Special Administrative Region, Hong Kong SAR, China; 2 The Pirbright Institute, Ash Road, Woking GU24 0NF, UK

**Keywords:** African swine fever, African swine fever virus, ASFV, wild boar, pig, full-length genome, genotype II, sequencing

## Abstract

This study represents the first report on the detection and whole-genome sequencing of African swine fever (ASF) viruses in wild boar in Hong Kong in 2021–2023. Wild boar samples collected via an ASF surveillance program by the Agriculture, Fisheries, and Conservation Department were tested for ASF viruses (ASFVs) using real-time polymerase chain reaction. ASF-positive carcasses were detected in four cases and hemadsorption, virus isolation, and whole-genome sequencing were conducted. The *B646L* gene, *E183L* gene, central variable region within the *B602L* gene, intergenic region between the *I73R* and *I329L* genes, *EP420R* gene, and multigene family members of the four ASFV strains were compared. The whole-genome phylogenetic relationships were studied. The comparative analysis of the genomes indicates that the ASFVs in these four cases have genetic similarities to Asian genotype II ASFVs, but are genetically distinct from each other, as well as the ASFV previously identified in a domestic pig farm in Hong Kong in 2021.

## 1. Introduction

African swine fever (ASF) is an infectious viral hemorrhagic disease that affects domestic pigs and wild boar. ASF is caused by the ASF virus (ASFV), which is the only member of the *Asfarviridae* family. ASFV has a large double-stranded DNA genome, with the genome size between 170 and 193 kbp, and composed of 150 to 200 open reading frames [1,2,3].

ASFV has been classified into 25 genotypes based on the partial sequence of the *B646L* gene, which encodes the capsid protein p72. Recent findings indicated that genotype XVIII represented a mixture of two distinct genotypes and proposed for its removal from the classification system [4]. A new classification system has been proposed recently in which all ASFV isolates are categorized into six genotypes by analyzing full-length p72 protein sequences [5]. An alternative classification system, which is based on comparisons of the entire encoded proteome, has also been proposed, allowing for the categorization of all available ASFV genomes into seven distinct biotypes [6].

ASFV, which was first described in Kenya, East Africa, in 1921, is endemic to sub-Saharan Africa, where all known ASFV genotypes have been identified [5,7,8]. The first introduction of ASFV to countries outside Africa occurred in 1957 and 1960 when ASFV genotype I spread to Europe [9]. Although largely eradicated from most countries by the mid-1990s, ASFV remains endemic on Sardinia Island [10]. In 2007, highly pathogenic ASFV genotype II was introduced from south-eastern Africa to Georgia and spread widely across Europe. This genotype subsequently emerged in Mainland China in 2018 and spread to other Asian countries in the following years [11,12]. In 2020, variants of genotype II with reduced virulence were detected in Heilongjiang and Hebei [13]. In the same year, genotype I ASFV, which caused chronic infection, was first reported in Guangxi, followed by reports in Shandong and Henan in 2021 [14,15]. By the end of that year and early 2022, a highly lethal recombinant ASFV comprising both genotypes I and II was identified in pig samples collected in Jiangsu, Henan, and the Inner Mongolia Autonomous Region. This recombinant virus was also reported in Vietnam and Russia in 2023 [16,17,18].

ASFVs have been detected in both domestic pigs and wild boar. While many ASF outbreaks reported in Asia involved domestic pigs, reports about ASF infections in wild boar have been made in Laos, Vietnam, Malaysia, Singapore, South Korea, and other parts of Asia in recent years [19,20,21,22]. The sources of infection include spillover from domestic pigs to wild boar, contaminated food waste, insect vectors, and environmental contamination [19,23,24].

The Hong Kong Special Administrative Region (SAR) is located in south-east China. Its northern land border connects with the south of Shenzhen, China, while the rest of the border is surrounded by water. The Hong Kong SAR territory is divided into three areas, namely Hong Kong Island, Kowloon, and the New Territories. About 1300–2500 native wild boar are scattered throughout the Hong Kong SAR [25,26]. They can be found in different habitats, such as forests and grasslands, and can very often be seen in human communities that are close to their habitats. These animals are attracted to human communities because of food sources, from unattended food scraps, garbage placed outdoors, illegal feeding, and vegetable farms. Other than wild boar, there are forty-three registered domestic pig farms in the Hong Kong SAR supplying pork to the city, together with the pork and pork products imported from all over the world. These registered domestic pig farms are located in the New Territories and are surrounded by habitats of wild boar.

As both wild boar and domestic pigs are susceptible to ASFV, these populations can be a source of infection to each other. Since 2019, a wild boar ASF surveillance program has been established by the Agriculture, Fisheries, and Conservation Department (AFCD) of the Hong Kong SAR with the goal to monitor the ASF situation in the wild boar population in the territory, and to inform actions to protect domestic pig farms. It encompasses passive and active surveillance. Dead wild boar found by the public will be removed by contractors employed by the government and suitable carcasses are tested for ASFV. While hunting activities are not conducted in the territory, the AFCD frequently mounts wild boar capture and dispatch operations to address nuisances to the public. Wild boar captured in these operations are sampled to test for ASFV by an official government laboratory, the Tai Lung Veterinary Laboratory, of the AFCD.

Several ASF outbreaks have been reported in the Hong Kong SAR. Three occasions of ASF were detected in imported domestic pigs in the city’s main slaughterhouse in 2019 [27,28]. The first domestic pig farm outbreak was detected in the territory in February 2021 [29], before the first case of ASF infection in wild boar in the Hong Kong SAR was detected later in the same year. Since then, sporadic cases of ASF infections in wild boar have been detected in the territory.

This study reports the first four cases of ASF in wild boar in the Hong Kong SAR in the period 2021–2023. Results of the laboratory diagnostics to identify the viruses and their characteristics, as well as details on whole-genome sequencing on the virus of each occasion, are described. Molecular epidemiology of these wild boar cases in the Hong Kong SAR and those reported in Asia are discussed. The analysis of this study indicates that the ASFVs in these four cases have genetic similarities to Asian genotype II ASFVs while being genetically distinct from each other and the ASFV previously identified in a domestic pig farm in the Hong Kong SAR in early 2021.

## 2. Materials and Methods

### 2.1. Sample Collection

In 2021–2023, a total of 80 wild boar (*Sus scrofa*) carcasses and blood samples of 532 captured wild boar from different districts of the Hong Kong SAR were tested for ASFV. The carcasses were initially found and reported by the public to the Hong Kong SAR government, and were subsequently attended by the animal carcass collection team as soon as possible. They were properly packaged to prevent contamination and delivered to the Tai Lung Veterinary Laboratory of the AFCD for ASFV diagnostic testing as part of the ASF surveillance program in the territory. Samples from carcasses including oronasal swabs, kidneys, spleens, tonsils, and lymph nodes were collected in a viral transport medium (medium 199 supplemented with 0.5% bovine serum albumin and antibiotics) during post-mortem examinations in the laboratory. Additionally, blood samples from captured wild boar were collected with sterile syringes, stored in EDTA vacutainer, and delivered to the AFCD for analysis.

### 2.2. Viral DNA Extraction, Real-Time PCR, and Initial ASFV Genotyping

DNA was extracted from oronasal swab, homogenized kidney, spleen, tonsil, lymph node, and blood samples using NucliSENS easyMAG (bioMérieux, Marcy-l’Étoile, France) according to the manufacturer’s instructions. Real-time polymerase chain reaction (RT-PCR) was performed using the primer set targeting the N-terminal conserved region of the *B646L* gene (p72) (forward: 5′-CTGCTCATGGTATCAATCTTATCGA-3′; reverse: 5′-GATACCACAAGATCRGCCGT-3′; probe: 5′-FAM-CCACGGGAGGAATACCAACCCAGTG-BHQ1-3′) as previously described [30,31]. For ASFV genotyping, 478 bp fragments corresponding to the C-terminal regions of the *B646* gene were amplified using the primers P72-U (5′-GGCACAAGTTCGGACATGT-3′) and P72-D (5′-GTACTGTAACGCAGCACAG-3′), as previously described [32]. The PCR amplicons were analyzed by gel electrophoresis and purified using a QIAquick PCR Purification kit (QIAGEN, Hilden, Germany) followed by Sanger sequencing.

### 2.3. Virus Isolation and Hemadsorption at the Pirbright Institute

Selected tissue and swab samples tested RT-PCR positive by the AFCD were further tested by virus isolation (VI) at The Pirbright Institute, United Kingdom. Samples with positive VI results were subsequently tested by hemadsorption (HAD). ASFV HAD titrations were performed using porcine bone marrow cells (PBMs) extracted from the long leg bones of 4-week-old uninfected pigs. The PBMs were seeded in 96-well plates at a density of 1.0–1.6 × 10^7^ cells/mL in Earle’s Balanced Salt Solution (EBSS) supplemented with 10% heat-inactivated porcine serum, 100 U/mL penicillin, 100 µg/mL streptomycin, and 1% HEPES solution, and incubated in a humidified chamber at 37 °C with 5% CO_2_ for 3 days. Homogenized tissue (*n* = 1) and swab samples (*n* = 2) underwent a series of ten-fold dilutions to inoculate the PBMs in freshly prepared EBSS containing 15% heat-inactivated porcine serum, 100 U/mL penicillin, and 100 µg/mL streptomycin. Samples were run in quadruplicate, and ASFV Malta 78 isolate was used as a positive control. Plates were incubated for 6 days, and results were calculated using the Spearman–Karber formula to express titers as log10 HAD50/mL. HAD-positive wells were pooled and centrifuged at 1500× *g* for 5 min. The supernatant was used to inoculate fresh PBMs in culture flasks containing EBSS with 15% heat-inactivated porcine serum, 100 U/mL penicillin, and 100 µg/mL streptomycin. Inoculated PBMs were incubated in a humidified chamber at 37 °C with 5% CO_2_ and harvested after 3 days once HAD was observed. Viral propagation was confirmed on the cell culture supernatant by performing ASFV real-time PCR described above.

### 2.4. Whole-Genome Sequencing and Analysis

One sample from each case was chosen for subsequent next-generation sequencing (NGS) using an Illumina sequencing protocol by the AFCD. Briefly, total DNA was quantified using Qubit Fluorometer (Thermo Fisher Scientific, Waltham, MA, USA). Nextera XT DNA library preparation kit (Illumina, San Diego, CA, USA) was used for preparing sequencing libraries according to the manufacturer’s instructions. The sequencing was performed on a MiSeq sequencing platform using MiSeq Reagent kit v3 (2 × 300 bp) (Illumina, San Diego, CA, USA). Adapter trimming and quality filtering of the raw sequencing data were performed using BBDuk (BBMap version 37.62) [33]. The processed sequencing reads were then mapped to the ASFV reference genomes using the Burrows–Wheeler Aligner (version 0.7.17) and Samtools (Version 2.0.4) [34,35]. Subsequently, the mapped reads were assembled using the SPAdes (Version 3.12.0) assembly tool and mapped to Georgia 2007/1 using the Burrows–Wheeler Aligner (Version 0.7.17) [36]. The consensus genome sequence was generated using iVar (Version 1.2.2) [37]. The location of protein-coding sequences was identified and annotated using the Genome Annotation Transfer Utility (GATU) software with Georgia 2007/1 as the reference genome [38]. The identity of the predicted protein sequences in the wild boar samples was compared to that of the Georgia 2007/1 strain using an in-house Python script (Python version 3.11.5) and manual verification. Average nucleotide identity (ANI) between ASFV genomes was calculated using FastANI (Version 1.1) [39].

### 2.5. Conventional PCR and Sanger Sequencing

Regions with ambiguous reads, including indels, homopolymer stretches, or mutations resulting in early truncation, were validated through PCR amplification and Sanger sequencing to confirm the next-generation sequencing results. The amplification of gene fragments was performed using Platinum SuperFi II DNA Polymerase (Thermo Fisher Scientific, Waltham, MA, USA) using the following PCR conditions: initial denaturation at 98 °C for 30 s, followed by 35 cycles each at 98 °C for 10 s, 60 °C for 10 s, 72 °C for 30 s, and a final extension at 72 °C for 5 min. Subsequently, PCR amplicons were analyzed by electrophoresis and purified using a QIAquick gel extraction kit (QIAGEN, Hilden, Germany) following the manufacturer’s instructions. Sanger sequencing was performed using the ABI3500 Genetic Analyzer (Applied Biosystems, Foster City, CA, USA) with a BigDye Terminator v3.1 Cycler Sequencing Kit and a BigDye XTerminator Purification Kit (Applied Biosystems, Foster City, CA, USA) according to the manufacturer’s instructions.

### 2.6. Phylogenetic Analysis

A total of 54 sequences of the ASFV *B646L* partial coding sequence (CDS) from various genotypes, along with the four ASFV strains identified from wild boar, were included in the phylogenetic analysis. The CDS corresponding to amino acid positions 499 to 631, based on the Georgia 2007/1 reference genome, was selected for this study. Multiple sequence alignment of the partial CDS from different ASFV strains and the four wild boar samples was performed using MAFFT v7.475 [40]. A maximum likelihood phylogenetic tree was constructed using MEGA X (Version 10.2.6), employing the Kimura 2-parameter model with a gamma distribution to account for rate variation among sites [41]. To assess the robustness and reliability of the inferred phylogenetic tree, 1000 bootstrap replications were conducted.

A total of 102 ASFV genomes identified in domestic pigs and wild boar from Asian, African, and European countries, along with the four ASFV strains identified from wild boar, were included in the phylogenetic analysis. The genome termini were excluded from the analysis, and only the regions from multigene family *(MGF) 360-1L* to *DP96R* were analyzed. Multiple nucleotide alignment was conducted using MAFFT v7.475 [42]. A maximum likelihood phylogenetic tree was constructed using IQ-TREE (Version 2.1.4-beta) [43]. The optimal substitution model was determined using ModelFinder [44]. An ultrafast bootstrap analysis with 10,000 replications was performed to assess branch support in the phylogenetic tree [45].

### 2.7. Recombination Analysis

The whole-genome sequences of the four ASFV strains identified in wild boar in this study, along with three genotype I ASFV strains (MZ945536.1 China_Pig/HeN/ZZ-P1/2021, MZ945537.1 China_Pig/SD/DY-I/2021, and AM712240.1 OURT 88/3 (avirulent_field_isolate)), as well as two genotype II ASFV strains (FR682468.2 Georgia_2007/1 and MK333180.1 China_Pig/HLJ/2018), were selected for multiple sequence alignment using MAFFT v7.475. Recombinant breakpoints were predicted using multiple methods with default settings in RDP4 software v4.101 [46,47,48,49,50,51,52]. Only potential recombination breakpoints predicted by at least four methods with a *p*-value < 0.05 were considered [53]. Additionally, a UPGMA (unweighted pair group method with arithmetic mean) tree was constructed, including these ASFV strains and three additional recombinant ASFV strains (OQ504954.1 China_Pig/Henan/123014/2022, OQ504955.1 China_Pig/Inner_Mongolia/DQDM/2022, and OQ504956.1 China_Pig/Jiangsu/LG/2021) previously reported in China, using Mega X (Version 10.2.6) [16]. To assess the robustness and reliability of the inferred phylogenetic tree, 1000 bootstrap replications were conducted.

## 3. Results

### 3.1. ASF-Positive Wild Boar Carcasses and Their Distributions

**Figure 1:**
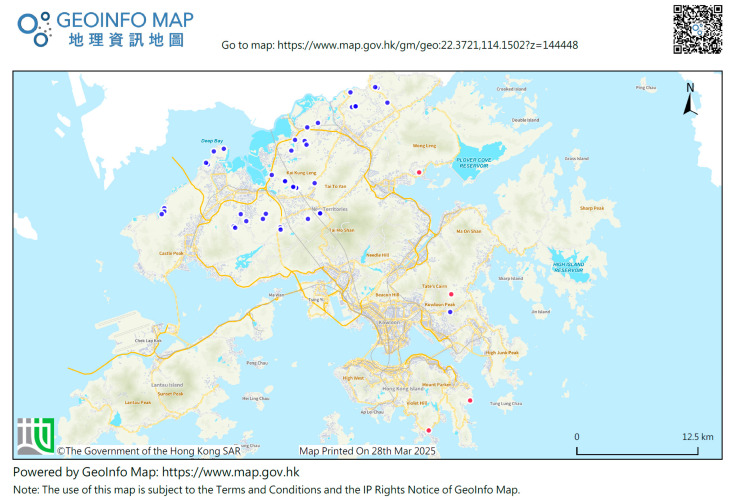
Locations of the African swine fever (ASF)-infected wild boar carcasses found in the Hong Kong Special Administrative Region (SAR) in 2021–2023 (red dots: locations of ASF-infected wild boar carcasses; blue dots: locations of domestic pig farms). The non-English term represents “Geoinfo Map.”

Out of the 80 wild boar carcasses and 532 blood samples from captured wild boar tested in 2021–2023, six carcasses were detected with ASFV. These six carcasses were involved in four different cases in four areas in the Hong Kong SAR: one in the vicinity of Cape Collinson Path, Siu Sai Wan, in September 2021; one in Wong Yue Tan, Tai Po, in January 2022; three at Stanley Village Road, Stanley, in February 2022; and one in Tai Lam Wu, Sai Kung, in May 2022. The locations of carcasses found are shown in Figure 1. No sample was detected with ASFV in 2023.

All tissues sampled tested positive for ASFV. Data of one carcass per case were selected to be presented in Table 1. The Ct values for these organ samples ranged from 17.07 to 25.97. Additionally, oronasal swabs collected from these cases yielded positive results, with Ct values ranging from 20.85 to 26.69.

### 3.2. ASFV p72 Genotyping, Virus Isolation, and HAD

Sequencing and phylogenetic analysis of the C-terminal end of the *B646L* gene showed that the ASFV strains obtained from the four wild boar were 100% identical to the ASFV previously identified in the Hong Kong SAR (HK202103) and clustered with the genotype II ASFVs reported in Europe and Asia (Supplementary Figure S1) [29]. ASFV was successfully isolated from the oronasal swab of ASFV/HKWB2022S-10414 and from the kidney and oronasal swab of ASFV/HKWB2022SK-13869, and the viruses were demonstrated to be hemadsorbing. The isolation of ASFV failed in the samples of ASFV/HKWB2021SSW-12112 and ASFV/HKWB2022TP-00522, as well as the spleen of ASFV/HKWB2022S-10414, which may be attributed to severe autolysis of the samples (Table 1).

### 3.3. Characterization of the Complete Genome Sequences of ASFVs

One sample with a lower Ct value was selected from each case and subjected to whole-genome sequencing. The length of the complete genomes of the four ASFV strains identified from wild boar ranged from 189,730 bp to 191,076 bp, with GC content from 38.36% to 38.40%, and 192 to 194 open reading frames (ORFs) were annotated using GATU. In the left variable region (LVR), an equal number of members from the multigene families (*MGF*s)—*MGF 360* (14 members), *MGF 505* (10 members), *MGF 300* (3 members), and *MGF 100* (1 member)—were identified in all four wild boar samples. Notably, while 12 members of *MGF 110* were identified in ASFV/HKWB2021SSW-12112 and ASFV/HKWB2022TP-00522, only 11 members were identified in ASFV/HKWB2022S-10414 and ASFV/HKWB2022SK-13869. *MGF 110-3L* was absent in ASFV/HKWB2022S-10414. Additionally, *MGF 110-13La* and *MGF 110-13Lb* were fused into a single *MGF 110-13L* in ASFV/HKWB2022SK-13869. Truncation of *MGF 110-7L* is found in all four ASFV strains. In the right variable region (RVR), all four strains contained the same number of members from the *MGF*s: *MGF 360* (5 members), *MGF 100* (2 members), and *MGF 505* (1 member) (Table 2). The ANI between the wild boar ASFV genomes ranged from 99.9768% to 99.9912%. Pairwise ANI comparing these strains to HK202103 and genotype II reference strain Georgia 2007/1 complete genomes are shown as a matrix of pairwise identity values in Supplementary Table S1.

To further discriminate the strains, regions of the *E183L* (p54) gene, the central variable region (CVR) of *B602L*, the tandem repeat sequences (TRS) within the intergenic region (IGR) between *I73R* and *I329L*, and the *EP402R* (CD2v) gene were extracted from the whole-genome sequences for comparison. The full-length *E183L* gene indicated that all four strains were identical to each other and belonged to genotype IIa. The tetrameric amino acid repeats within the CVR were found to be 100% identical to those of strain HK202103, Pig/HLJ/2018 (the first reported ASF case in Mainland China), China/GD/2019 (an isolate collected in Guangdong), and Georgia 2007/1, containing ten tandem amino acid repeat units (BNDBNDBNAA), which correspond to CVR1. The TRS within the IGR between *I73R* and *I329L* were also 100% identical among the four strains and to strains HK202103, Pig/HLJ/2018, and China/GD/2019; however, they exhibited a 10-nucleotide insertion compared to Georgia 2007/1, corresponding to IGR II. Additionally, phylogenetic analysis of the *EP402R* (CD2v) gene revealed that all four strains belonged to serotype 8.

Among the four sequenced ASFV strains, several notable single-nucleotide polymorphisms (SNPs) resulting in nonsynonymous substitutions were identified in specific CDS across the strains. These mutations were strain-specific, not having been previously observed in other available genotype II ASFV genomes. For instance, unique SNPs were identified in the *EP1242L* and *M448R* CDS of strain ASFV/HKWB2021SSW-12112. In strain ASFV/HKWB2022TP-00522, SNPs were identified in *P1192R*, *I243L*, and *EP153R* CDS. Strain ASFV/HKWB2022S-10414 had SNPs identified in the *MGF 110-2L*, *MGF 360-4L*, *DP96R*, and *C962R* CDS. Additionally, a 719 bp deletion compared to the Georgia 2007/1 strain and other Genotype II ASFV strains was observed, leading to the truncation of *MGF 110-3L* and *ASFV G ACD 00120* CDS. SNPs were also identified in *MGF 360-15R*, *B646L*, *B475L*, and *D1133L* CDS of strain ASFV/HKWB2022SK-13869 (Table 3).

### 3.4. Whole-Genome Phylogenetic Analysis

**Figure 2:**
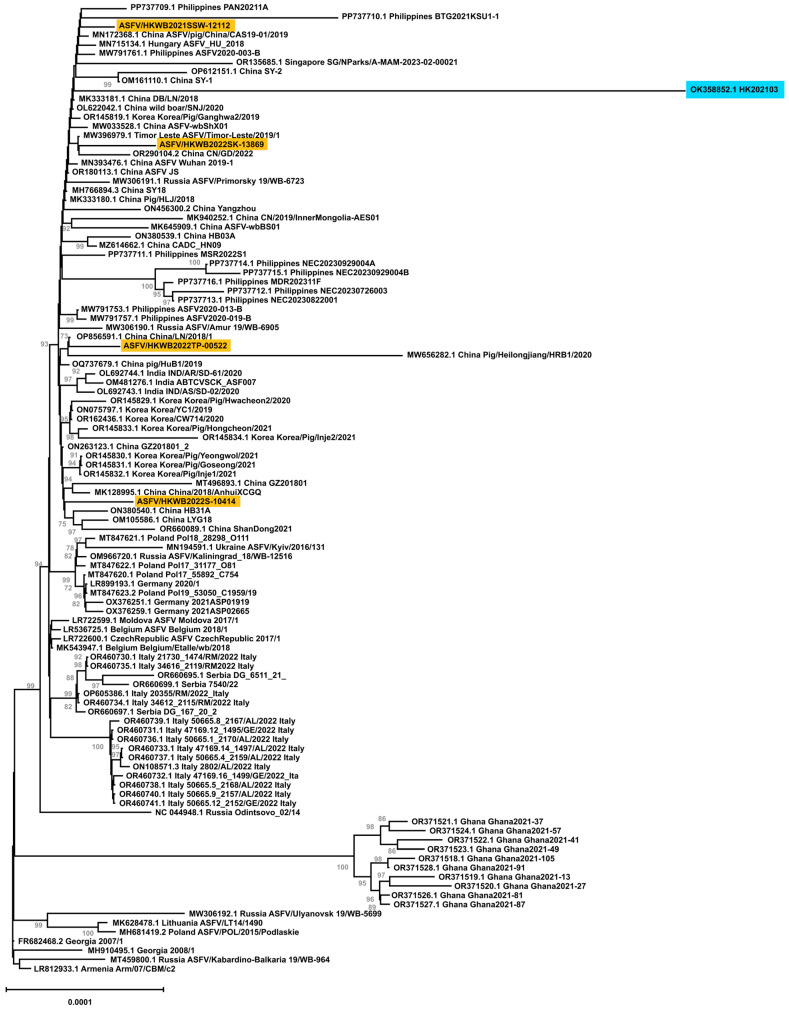
Phylogenetic analysis of whole-genome sequences from four ASFV strains identified in wild boar in the Hong Kong SAR during 2021–2023 and other genotype II ASFV strains. ASFVs identified in this study are highlighted in orange, while the previously identified ASFV in a domestic pig farm in the Hong Kong SAR is highlighted in blue. Only bootstrap values greater than 70 are shown.

A phylogenetic tree was constructed to study the phylogenetic relationships among genotype II ASFVs identified in Asian and European countries (Figure 2). Considering the uncertainty of the terminal inverted repeat sequence and the length variations in the genome termini, only the regions from *MGF 360-1L* to *DP96R*, which is approximately 185,155 bp including gaps, were included in the analysis. Complete genomes collected from domestic pigs (50 genomes) and wild boar (52 genomes) from 21 countries/places including the Hong Kong SAR (1), Mainland China (25), Armenia (1), Belgium (2), the Czech Republic (1), Georgia (2), Germany (3), Hungary (1), India (3), Italy (14), South Korea (9), Lithuania (1), Moldova (1), Philippines (11), Poland (5), Russia (6), Singapore (1), Timor Leste (1), Serbia (3), Ghana (10), and Ukraine (1) from 2007 to 2023 were downloaded from the Genbank and included in the phylogenetic analysis.

The four strains identified from wild boar clustered within the subgroup primarily composed of strains identified in Asia including Mainland China, South Korea, India, Philippines, Timor Leste, and Singapore as well as a strain identified in Hungary in 2018. Notably, the four strains did not cluster with each other in the phylogenetic tree. None of these four strains clustered with the ASFVs identified in wild boar in other countries. Strain ASFV/HKWB2022TP-00522 clustered with China/LN/2018/1 (OP856591), which was collected in a domestic pig in Mainland China in 2018 with a bootstrap value of 73%. Strain ASFV/HKWB2022SK-13869, ASFV/HKWB2022S-10414, and ASFV/HKWB2021SSW-12112 did not cluster with a specific strain due to low bootstrap values (<70%). It is also noted that these four strains were found to be distantly related to the ASFV strain HK202103, which was previously identified in a domestic pig in a farm in the Hong Kong SAR, indicating that the sources of these ASFVs in the Hong Kong SAR were different, and the spread of the disease in wild boar was not likely to be caused by this domestic pig farm. 

### 3.5. Recombination Analysis of the Four ASFV Strains Identified from Wild Boar

By comparing with the selected genotype I and genotype II ASFV strains, no recombinant breakpoints were detected in these four ASFV strains identified from wild boar. These four strains clustered with other genotype II strains in the UPGMA tree (Supplementary Figure S2), while the three recombinant ASFV strains were grouped in a separate cluster.

### 3.6. Data Availability

The full-length genome sequences of the four ASF cases (ASFV/HKWB2021SSW-12112, ASFV/HKWB2022TP-00522, ASFV/HKWB2022S-10414, ASFV/HKWB2022SK-13869) have been deposited in the GenBank database under the accession numbers PV400254, PV400255, PV400256, and PV400257, respectively.

## 4. Discussion

The first case of ASF in Mainland China was identified in August 2018, after which the disease rapidly spread to multiple provinces across the country [67]. Although the number of reported ASFV-infected wild boar carcasses in Mainland China is limited compared to domestic pigs, research has indicated that wild boar play a crucial role in the maintenance and transmission of ASFV, particularly in Asia [68,69]. Given the potential for prolonged virus circulation and the close geographical proximity between Guangdong and the Hong Kong SAR, a better understanding of the ASF disease status in wild boar populations is paramount for comprehensive control efforts, particularly following the ASF outbreaks reported in Guangdong in 2019 [70,71]. A wild boar ASF surveillance program in the Hong Kong SAR was implemented in late 2019, which aimed at monitoring the ASFV status in wild boar populations and mitigating the risk of ASF transmission to domestic pigs.

During the surveillance period, one outbreak of ASFV was detected in a local domestic pig farm located in the New Territories of the Hong Kong SAR in early Feburary 2021. Ten domestic pigs were tested postive for ASFV, leading to a culling conducted in mid-Feburary 2021 [29]. Following the culling operation, no new outbreaks were reported from local farms, and no wild boar were found to be ASFV-positive. In September 2021, a wild boar carcass collected from Siu Sai Wan tested positive for ASFV. Subsequently, in January, February, and May 2022, ASFV was identified in wild boar carcasses collected from Tai Po, Stanley, and Sai Kung, respectively.

The initial genotyping of the ASFV strains identified from wild boar was performed by sequencing the C-terminal end of *B646L* (p72) gene. The partial *B646L* sequence indicated that all four ASFVs identified in wild boar belonged to p72 genotype II. Further discrimination into subgroups of closely related viruses was conducted through sequence analysis of the full-length *E183L* (p54) gene, the CVR within the *B602L* gene, and the IGR between the *I73R* and *I329L* genes [72,73]. The *E183L* genotyping confirmed that these strains are classified as p54 genotype IIa, consistent with other genotype II strains found in Mainland China, while p54 genotypes IIb and IIc have only been identified in Africa to date [15,74]. Analysis of the CVR sequences indicated that the CVR of the four ASFVs were identical and belonged to CVR1. Currently, over ten CVR variants have been reported in genotype II ASFVs in the literature. Specifically, five CVR variants were documented in Mainland China [15,75,76,77]. The CVR1 variant presented in the four ASFVs identified in wild boar is the most dominant variant among genotype II ASFVs in Europe and Asia [75,78,79]. The analysis of the IGR sequences showed that these four ASFVs were identical and belonged to IGR II variant. Currently, four IGR variants are circulating in Europe and Asia [75,78,80,81,82]. The IGR I variant, present in the Georgia 2007/1 strain, has been circulating in Vietnam, South Korea, Russia, and detected in the wild boar in Mainland China and Poland [75,78,80,81,82]. The IGR II variant is now the predominant IGR variant found in domestic pigs in Europe and Asia [72,78]. The IGR III variant was first discovered in Mainland China in 2019 and has since been reported in South Korea, Poland, and Vietnam [15,75,80,83]. Lastly, the IGR IV variant was discovered exclusively in Poland in 2019 and 2020, and subsequently in Vietnam in 2023 [84,85]. Among the three IGR variants circulating in Mainland China, our data showed that the four ASFVs identified in wild boar belonged to the IGR II variant, which is the most dominant variant circulating in Asia.

The *EP402R* gene, which encodes the CD2v protein, is essential for the adsorption of red blood cells to virus-infected cells and extracellular virus particles [86]. Beyond its role in hemadsorption, the complete *EP402R* sequence is valuable for differentiating ASFV strains from various regions [87]. In 2020, a lower virulence strain of genotype II emerged in Mainland China, marked by mutations or deletions in the *EP402R* gene that resulted in a non-hemadsorbing phenotype [13,88]. Due to unsuccessful virus isolation in most of the selected samples tested in this study, hemadsorption assays were not performed on them. Consequently, the *EP402R* gene sequence was extracted from whole-genome sequencing data to investigate potential mutations that may correlate with virulence and to assess whether the *EP402R* sequence could further differentiate these four strains identified from wild boar. Our data demonstrated that the *EP402R* sequences in the four strains were identical to each other and belonged to serotype 8, which is the dominant serotype among genotype II ASFVs in Mainland China [15].

Our data indicated that the four strains identified from wild boar could not be differentiated by the commonly used genotypic markers. This finding was consistent with previous observations that Asian and European ASFV genotype II strain exhibited a high nucleotide similarity of 99.99%, which suggested limited genetic diversity within this group [89]. Similarly, another study found that the ASFVs within the Eastern Asia Lineage were relatively homogeneous compared to the prototype strain HLJ/2018 [12]. These findings collectively suggested that the current classification and differentiation of ASFV based on limited gene subsets is insufficient for accurately distinguishing closely related strains. To enhance resolution and effectively differentiate these four strains, a whole-genome sequencing approach was adopted in this study.

In the whole-genome phylogenetic analysis, the genome termini were excluded due to variablity in length among the analyzed ASFV genomes. The regions spanning from *MGF 360-1L* to *DP96R* were included in the analysis, encompassing both coding and non-coding sequences. This approach allowed for the inclusion of mutations within intergenic regions, as well as the inclusion of large fragment deletions in the analysis. Whole-genome phylogenetic analysis of ASFV genotype II genomes showed the four strains identified from wild boar clustered within the Asian Lineage. Strain ASFV/HKWB2022TP-00522 was clustered with China/LN/2018/1 (OP856591), which was found in a domestic pig in Mainland China in 2018; however, strain ASFV/HKWB2022SK-13869, ASFV/HKWB2022S-10414, and ASFV/HKWB2021SSW-12112 did not cluster with a specific strain due to low bootstrap values. There was no evident clustering of the four strains in our study with the ASFVs identified in other wild boar, nor was there any significant clustering among the four strains. The low bootstrap value might have resulted from the small amount of phylogenetic signal, leading to short internal tree branches that were difficult to resolve, or informative sites in the genome that were insufficient to generate a phylogenetic tree with high support [90]. No evidence was found to support any epidemiological linkage between these strains and the ASFV strains identified from other wild boar in the whole-genome phylogenetic analysis. Additionally, given the overlapping time period of the reported recombinant ASFV strains in China, we investigated the potential recombination connection between these strains and the four ASFV strains identified from wild boar. Our analysis showed no strong evidence that these four ASFV strains share recombination events with the recombinant ASFV strains previously reported in Mainland China, as no recombination was detected in them, which clustered distinctly within Genotype II.

Pairwise comparison of the whole-genome sequences of the four strains identified from wild boar showed an ANI ranging from 99.9768% to 99.9912% (Supplementary Table S1), suggesting that they likely originated from a common ancestor. To further enhance the resolution in distinguishing these closely related strains and to assess their genetic diversity, molecular epidemiology analyses focusing on SNPs and indels in coding regions were conducted. The analysis revealed notable SNPs and indels in coding regions among the genomes of the four ASFV strains, specifically within essential genes and *MGF* (Table 3). Among the five *MGF*s, gene deletions and deleterious mutations in the four strains were primarily located in the *MGF 110* and *MGF 360* members, similar to the deletions observed in both the China/GD/2019 strain and ASFV_YNFN202103 strain, which were isolated from a domestic pig in Guangdong and Yunnan, respectively [71,88].

Further analysis on the CDS revealed notable mutations in individual strains compared to the genotype II reference strain Georgia 2007/1 (Table 3). A 719 bp fragment deletion, compared to the Georgia 2007/1 strain, resulted in the truncation of both *MGF 110-3L* and *ASFV G ACD 00120* CDS in strain ASFV/HKWB2022S-10414. This deletion has not been reported in other genotype II ASFVs. A similar deletion was reported in Tanzania/Rukwa/2017/1 (LR813622.1) and TAN/17/Kibaha (ON409979.1) where a 678 bp deletion, compared to the Georgia 2007/1 strain, led to the truncation of three genes (*MGF 110-3L-4L* and *ASFV G ACD 00120*) [91]. In addition to the 719 bp deletion, novel mutations involving nonsynonymous substitutions were also identified in this strain, including *C962R*, which likely plays a role in base excision repair or other repair pathways in ASFV [63]; *DP96R*, a virulence-associated protein involved in the suppression of type I interferon production [62]; and *MGF 360-4L* and *MGF 110-2L*, both of which are involved in virus cell tropism [55]. Novel mutations in the essential genes were also identified in the other three strains. Nonsynonymous substitutions were found in the DNA topoisomerase type II-encoding gene *P1192R*, the transcription factor SII-encoding gene *I243L*, and the *EP153R* type II transmembrane protein in strain ASFV/HKWB2022TP-00522. In strain ASFV/HKWB2022SK-13869, nonsynonymous substitutions were detected in the p72 capsid protein-encoding gene *B646L* [55], the *D1133L* putative helicase-encoding gene [65], the *B475L* gene, which is potentially involved in evasion of host antiviral immunity [64], and the *MGF 360-15R* gene, which is associated with virus cell tropism [55]. Additionally, strain ASFV/HKWB2021SSW-12112 contained nonsynonymous substitutions in the *EP1242L* gene, which is involved in viral gene transcription [41], and the *M448R* gene, which is a T-cell antigen with protective potential [57].

In addition to nonsynonymous substitutions, deletions in the *MGF* genes were observed in the four ASFV strains, resulting in either formation of a fusion gene or truncation of a gene into two separate genes. The deletions in the poly-G region in the *MGF 110-10L—MGF 110-14L* fusion CDS in the four strains resulted in the frame shift and early truncation of the protein, resulting in two separate CDS, namely *MGF 110-11L* and *MGF 110-14L*. The *MGF 110-11L* CDS were identical among all the four strains analyzed and exhibited 100% identity with previously reported strains, including Pig/HLJ/18 and other ASFV strains reported in Asian and European countries. In contrast, the *MGF 110-14L* CDS displayed more variability among the four strains. Notably, the *MGF 110-14L* CDS in strains ASFV/HKWB2021SSW-12112 and ASFV/HKWB2022S-10414 were identical to each other and also exhibited 100% identity with other ASFV strains reported in Mainland China, India, Vietnam, Russia, Ukraine, Georgia, Tanzania, and Poland. Strain ASFV/HKWB2022TP-00522 contained one fewer glycine residue in its *MGF 110-14L* predicted protein compared to strains ASFV/HKWB2021SSW-12112 and ASFV/HKWB2022S-10414, and it was identical to the ones identified in Italy (accession number OR460732.1). Additionally, the *MGF 110-14L* in strain ASFV/HKWB2022SK-13869 was characterized by the presence of extra amino acid residues at its C-terminal end, with its sequence being identical to those collected from strains in Vietnam (accession number MW465755.1), Mainland China (accession numbers MW656282.1, ON263123.1, OR126359.1), Russia (accession number MW306191.1), and Italy (accession numbers OP605386.1, OR460730.1, OR460731.1, OR460733.1, OR460735.1, OR460737.1, OR460739.1, OR460740.1, OR460741.1). For *MGF 110-13Lb* CDS, a single-nucleotide deletion in this gene in strain ASFV/HKWB2022SK-13869 was observed, which resulted in frame shift and fusion with the downstream *MGF 110-13La* CDS as *MGF 110-13L*. The resulting fusion CDS has been identified in the ASFV reported in Vietnam (accession number MW465755.1), Poland (accession numbers MG939583.1, MT847620.1, MT847621.1, MT847622.1, MT847623.2), Mainland China (accession numbers MK128995.1, MK333181.1, MK645909.1, MN172368.1, MT496893.1, MW656282.1, MW033528.1, OM161110.1, OP612151.1, OL622042.1, MH766894.3), Timor-Leste (accession number MW396979.1), and Russia (accession numbers OM966715.1, OM966716.1, OM799941.1) as well as in the Pig/HLJ/18 strain (accession number MK333180.1). Although these wild boar ASFVs shared high nucleotide similarity, the presence of SNPs and indels in specific strains suggested that these viruses were not directly epidemiologically linked and have diverged during circulating. It is speculated that these four ASFV strains were introduced from multiple sources rather than being epidemiologically connected.

The first ASFV outbreak in a domestic pig farm in the Hong Kong SAR was reported in 2021 located in the New Territories. The identified strain, HK202103, belonged to genotype II and is characterized as the IGR (*I73R-I329L*) II variant, CVR-1, serogroup 8 [29]. In this study, the relationship between the four ASFV strains identified from wild boar and HK202103 was investigated using genomic data to determine whether these strains are derived from or transmitted by HK202103. Results showed that the ANI between the ASFV genomes from wild boar and HK202103 ranged from 99.9659% to 99.9773% (Supplementary Table S1). The sequences of the *B646L* gene, IGR, CVR gene, and *EP402R* gene from the four ASFV strains identified from wild boar were found to be 100% identical to those of HK202103. However, the whole-genome phylogenetic analysis revealed no distinct clustering among the four strains with HK202103 due to low bootstrap values. Instead, both the strains identified from wild boar and HK202103 were clustered within the Asian Lineage. The low bootstrap value in the phylogenetic analysis may be attributed to a limited phylogenetic signal and insufficient informative sites in the genomic data [90]. Further molecular epidemiology analyses focusing on SNPs and indels revealed a range of mutations and genetic variations presented in the specific strains identified from wild boar but absent in HK202103, suggesting that these ASFVs underwent genetic drift during ciruclation and further suggesting that the sources of these ASFVs were not directly linked to HK202103, and the spread of ASFV in wild boar was unlikely to have resulted from the transmission from this domestic pig farm.

Based on the analysis of this report, the ASFV strains identified from wild boar in 2021–2023 were not epidemiologically connected with each other, nor with the first and only ASFV strain detected in a local pig farm in the Hong Kong SAR in 2021. Other additional domestic farm and wild boar cases did not occur until 2023 and 2024, respectively. Considering the temporal and geographical factors relating to the ASFVs detected at the time, it is suggested that there was a series of separate introductions of the disease to the wild boar population. The most likely possible introduction would be from contaminated meat. Despite the measures taken to prevent illegal feeding and access to food waste by scavenging, the chance of these boar making contact with contaminated meat is still present [92]. It is documented that ASFV has been found in contaminated pork and pork products [93,94,95]. Since ASF has been introduced to Asia, there was evidence from other Asian countries that ASFV is present in the human food chain [96,97,98,99]. Pork and pork products carried across borders can be a source of introduction of the virus. The locations of the four wild boar carcasses were close to urban areas, hence it was very likely that these cases were caused by contaminated meat through human activities.

This study represents the first report on the detection and whole-genome sequencing of ASFVs in wild boar in the Hong Kong SAR in 2021–2023. The comparative analysis of the genomes indicates that these ASFVs have genetic similarities to Asian genotype II ASFVs, but genetically distinct from each other as well as the ASFV previously identified in a domestic pig farm in the Hong Kong SAR in 2021. Based on our analysis, the source of infection in these wild boar remains unidentified. Further investigation is needed to determine whether these wild boar were infected through contact with contaminated food from nearby urban areas. Additionally, functional studies of the identified nonsynonymous mutations may provide insights into the mechanisms of virulence and immune evasion.

## Figures and Tables

**Table 1:** Virology results of the wild boar tested ASF virus (ASFV)-positive in the Hong Kong SAR in 2021–2023.

Case	Date	Location Found	Weight (kg)	Sample Type	RT-PCR (Ct)	Gel PCR	VI	HAD
ASFV/HKWB2021SSW-12112	2 September 2021	Siu Sai Wan	55	Spleen	18.48	Positive	Not Isolated	-
				Tonsil ^#^	17.89	Positive	Not Isolated	-
				Lymph node	18.31	Positive	Not Isolated	-
				Kidney	19.11	Positive	Not Isolated	-
				Oronasal swab	25.86	-	Not Isolated	-
ASFV/HKWB2022TP-00522	12 January 2022	Tai Po	29	Spleen ^#^	19.33	Positive	Not Isolated	-
				Tonsil	25.97	Positive	-	-
				Lymph node	20.85	Positive	Not Isolated	-
				Kidney	21.02	Positive	-	-
				Oronasal swab	26.69	Positive	Not Isolated	-
ASFV/HKWB2022S-10414	24 February 2022	Stanley	74	Spleen ^#^	17.14	Positive	Not Isolated	-
				Tonsil	23.96	Positive	-	-
				Lymph node	17.07	Positive	-	-
				Kidney	21.94	Positive	-	-
				Oronasal swab	20.85	Positive	Positive	Positive
ASFV/HKWB2022SK-13869	27 May 2022	Sai Kung	43	Spleen	19.73	Positive	-	-
				Tonsil	18.80	Positive	-	-
				Lymph node	20.02	Positive	-	-
				Kidney ^#^	17.95	Positive	Positive	Positive
				Oronasal swab	22.02	-	Positive	Positive

^#^ Sample selected for whole-genome sequencing. “-” indicates that the test was not performed.

**Table 2:** Characterization of four ASFV genomes identified in wild boar.

Strain	Genome Length (bp)	GC Content (%)	Number of ORFs	Number of *MGF* Members							
				LVR					RVR		
				*MGF 360*	*MGF 110*	*MGF 505*	*MGF 300*	*MGF 100*	*MGF 360*	*MGF 100*	*MGF 505*
ASFV/HKWB2021SSW-12112	190,165	38.40	194	1	12 *	3	14	9	2	5	1
ASFV/HKWB2022TP-00522	190,181	38.40	194	1	12 *	3	14	9	2	5	1
ASFV/HKWB2022S-10414	189,730	38.39	192	1	11 *	3	14	9	2	5	1
ASFV/HKWB2022SK-13869	191,076	38.36	193	1	11 *	3	14	9	2	5	1

* Note: *MGF 110-3L* is absent in ASFV/HKWB2022S-10414, and *MGF 110-13La* and *MGF 110-13Lb* have been combined into 13L in ASFV/HKWB2022SK-13869. Truncation of *MGF 110-7L* is found in all ASFV strains of these wild boar.

**Table 3:** Comparison of the coding sequences (CDSs) of four ASFV genomes identified in wild boar.

Strain	Gene	Function	CDS Position *	Polymorphism Type	Change	Effect	Reference
ASFV/HKWB2021SSW-12112	*MGF 110-1L* CDS	Unknown	590	Substitution	G>A	Nonsense mutation	
	*MGF 110-7L* CDS	Induces host cell translation suppression and stress granule formation	170–183	Deletion	TGTGAAGATGGGAT	Frame shift and early truncation	[33]
	*MGF 110-10L—MGF110-14L* fusion	Unknown	346–347	Deletion	GG	Frame shift and early truncation.	
	*MGF 360-10L* CDS	Modulates type I interferon response and is associated with viral virulence	986	Substitution	A>G	Nonsynonymous substitution (N329S)	[54]
	*MGF 360-14L* CDS	Involved in virus cell tropism; may be required for efficient virus replication in macrophages	845–846	Insertion	G	Frame shift and early truncation	[55]
	*MGF 505-9R* CDS	Unknown	967	Substitution	A>G	Nonsynonymous substitution (K323E)	
	*ASFV G ACD 00190* CDS	Unknown	13	Deletion	T	Frame shift and early truncation	
	*ASFV G ACD 00350* CDS	Unknown	37–42	Deletion	GGGGGG	In-frame deletion	
	*EP1242L* CDS	Involved in viral gene transcription	2767	Substitution	G>A	Nonsynonymous substitution (V923I)	[41]
	*NP419L* CDS	DNA ligase; potentially involved in repair mechanisms	1241	Substitution	A>G	Nonsynonymous substitution (N414S)	[56]
	*DP60R* CDS	Unknown	53–54	Insertion	A	Frame shift and extension	
	*M448R* CDS	T-cell antigen with protective potential	163	Substitution	G>A	Nonsynonymous substitution (E55K)	[57]
	*I267L* CDS	Acts as a virulence factor by inhibiting RNA polymerase III-RIG-I-mediated immunity	583	Substitution	A>T	Nonsynonymous substitution (I195F)	[58]
ASFV/HKWB2022TP-00522	*MGF 110-1L* CDS	Unknown	590	Substitution	G>A	Nonsense mutation	
	*MGF 110-4L* CDS	Involved in virion assembly	260	Substitution	A>G	Nonsynonymous substitution (Q87R)	[59]
	*MGF 110-7L* CDS	Induces host cell translation suppression and stress granule formation	110	Deletion	C	Frame shift and early truncation	[33]
	*MGF 110-10L—MGF110-14L* fusion	Unknown	343–347	Deletion	GGGGG	Frame shift and early truncation.	
	*MGF 360-1La* CDS	Unknown	363	Substitution	T>G	Nonsynonymous substitution (S121R)	
	*MGF 360-10L* CDS	Modulates type I interferon response and is associated with viral virulence	986	Substitution	A>G	Nonsynonymous substitution (N329S)	[54]
	*MGF 505-9R* CDS	Unknown	967	Substitution	A>G	Nonsynonymous substitution (K323E)	
	*ASFV G ACD 00190* CDS	Unknown	13	Deletion	T	Frame shift and early truncation	
	*ASFV G ACD 00350* CDS	Unknown	37–42	Deletion	GGGGGG	In-frame deletion	
	*NP419L* CDS	DNA ligase; potentially involved in repair mechanisms	1241	Substitution	A>G	Nonsynonymous substitution (N414S)	[56]
	*DP60R* CDS	Unknown	53–54	Insertion	A	Frame shift and extension	
	*I267L* CDS	Acts as a virulence factor by inhibiting RNA polymerase III-RIG-I-mediated immunity	583	Substitution	A>T	Nonsynonymous substitution (I195F)	[58]
	*P1192R* CDS	Topoisomerase; involved in virus transcription and replication	645–648	Substitution	GGCG>AACA	Nonsynonymous substitution (A216T)	[60]
	*I243L* CDS	Transcription factor	506	Substitution	G>C	Nonsynonymous substitution (G169A)	[41]
	*EP153R* CDS	Involved in hemadsorption	421	Substitution	A>G	Nonsynonymous substitution (S141G)	[61]
ASFV/HKWB2022S-10414	*MGF 110-1L* CDS	Unknown	590	Substitution	G>A	Nonsense mutation	
	*MGF 110-2L* CDS	Involved in virus cell tropism; may be required for efficient virus replication in macrophages	5	Substitution	G>A	R2K	[55]
	*MGF 110-3L* CDS	Unknown	Entire CDS	Deletion	Deletion	Gene deletion	
	*MGF 110-7L* CDS	Induces host cell translation suppression and stress granule formation	110–111	Insertion	C	Frame shift and early truncation	[33]
	*MGF 110-10L—MGF110-14L* fusion	Unknown	346–347	Deletion	GG	Frame shift and early truncation.	
	*MGF 110-13Lb* CDS	Unknown	359–366	Deletion	GGGGGGGG	Frame shift and extension	
	*MGF 360-1La* CDS	Unknown	94	Substitution	G>A	Nonsynonymous substitution (E32K)	
			463	Substitution	G>A	Nonsynonymous substitution (E155K)	
	*MGF 360-4L* CDS	Involved in virus cell tropism; may be required for efficient virus replication in macrophages	574	Substitution	G>A	Nonsynonymous substitution (A192T)	[55]
	*MGF 360-19Ra* CDS	Involved in virus cell tropism; may be required for efficient virus replication in macrophages	355	Substitution	C>T	Nonsynonymous substitution (L119F)	[55]
			452	Substitution	C>T	Nonsynonymous substitution (P151L)	
			691	Substitution	C>T	Nonsynonymous substitution (H231Y)	
	*MGF 360-10L* CDS	Modulates type I interferon response and is associated with viral virulence	986	Substitution	A>G	Nonsynonymous substitution (N329S)	[54]
	*MGF 505-9R* CDS	Unknown	967	Substitution	A>G	Nonsynonymous substitution (K323E)	
	*ASFV G ACD 00120* CDS	Unknown	1–155	Deletion	Deletion	Gene deletion	
	*ASFV G ACD 00190* CDS	Unknown	13	Deletion	T	Frame shift and early truncation	
	*NP419L* CDS	DNA ligase; potentially involved in repair mechanisms	1241	Substitution	A>G	Nonsynonymous substitution (N414S)	[56]
	*DP60R* CDS	Unknown	53–54	Insertion	A	Frame shift and extension	
	*DP96R* CDS	Uridine kinase; inhibits interferons production and associated with virus virulence	196	Substitution	C>T	Nonsynonymous substitution (P66S)	[62]
	*I267L* CDS	Acts as a virulence factor by inhibiting RNA polymerase III-RIG-I-mediated immunity	583	Substitution	A>T	Nonsynonymous substitution (I195F)	[58]
	*C962R*	Potentially involved in repair mechanisms	1538	Substitution	G>A	Nonsynonymous substitution (R513H)	[63]
	*A238L* CDS	Downregulates host inflammatory responses	394	Substitution	G>A	Nonsynonymous substitution (A132T)	[42]
ASFV/HKWB2022SK-13869	*MGF 110-1L* CDS	Unknown	590	Substitution	G>A	Nonsense mutation	
	*MGF 110-3L* CDS	Unknown	272	Substitution	G>A	Nonsynonymous substitution (G91D)	
	*MGF 110-7L* CDS	Induces host cell translation suppression and stress granule formation	110–111	Insertion	C	Frame shift and early truncation	[33]
	*MGF 110-13Lb* CDS	Unknown	366	Deletion	G	Frame shift and fusion with *MGF 110-13 La*	
	*MGF 110-4L* CDS	Involved in virion assembly	325	Substitution	A>G	Nonsynonymous substitution (N109D)	[59]
	*MGF 110-10L—MGF110-14L* fusion	Unknown	344–347	Deletion	GGGG	Frame shift and early truncation.	
	*MGF 360-10L* CDS	Modulates type I interferon response and is associated with viral virulence	986	Substitution	A>G	Nonsynonymous substitution (N329S)	[54]
	*MGF 360-14L* CDS	Involved in virus cell tropism; may be required for efficient virus replication in macrophages	845–846	Insertion	G	Frame shift and early truncation	[55]
	*MGF 360-15R* CDS	Involved in virus cell tropism; may be required for efficient virus replication in macrophages	697	Substitution	G>A	Nonsynonymous substitution (A233T)	[55]
	*MGF 505-9R* CDS	Unknown	967	Substitution	A>G	Nonsynonymous substitution (K323E)	
	*ASFV G ACD 00190* CDS	Unknown	13	Deletion	T	Frame shift and early truncation	
	*ASFV G ACD 00350* CDS	Unknown	39–42	Deletion	GGGG	In-frame deletion	
	*NP419L* CDS	DNA ligase; potentially involved in repair mechanisms	1241	Substitution	A>G	Nonsynonymous substitution (N414S)	[56]
	*DP60R* CDS	Unknown	53–54	Insertion	A	Frame shift and extension	
	*I267L* CDS	Acts as a virulence factor by inhibiting RNA polymerase III-RIG-I-mediated immunity	583	Substitution	A>T	Nonsynonymous substitution (I195F)	[58]
	*B646L* CDS	Viral capsid	1175	Substitution	G>A	Nonsynonymous substitution (R392H)	[55]
	*B475L* CDS	Potentially involved in the host antiviral innate immunity evasion	853	Substitution	G>A	Nonsynonymous substitution (E285K)	[64]
	*D1133L* CDS	Helicase; potentially involved in viral replication	13	Substitution	G>A	Nonsynonymous substitution (E5K)	[65]
	*E199L* CDS	Involved in virus entry and induce autophagy	374	Substitution	A>T	Nonsynonymous substitution (E125V)	[66]

* The CDS position with reference to Georgia 2007/1.

## Data Availability

The full-length genome sequences of ASFV/HKWB2021SSW-12112, ASFV/HKWB2022TP-00522, ASFV/HKWB2022S-10414, and ASFV/HKWB2022SK-13869 have been deposited in the GenBank database under the accession numbers PV400254, PV400255, PV400256, and PV400257, respectively.

## Supplementary Materials

The following supporting information can be downloaded at: https://www.mdpi.com/article/10.3390/v17070896/s1; Figure S1: P72 genotype tree; Figure S2: UPGMA tree; Table S1: Comparison of average nucleotide identity (ANI) among four ASFV strains identified from wild boar, the strain HK202103 (OK358852.1) previously identified in a domestic pig on a farm in the Hong Kong SAR, and the reference strain Georgia 2007/1.
