# Supplementary material for: The First African Swine Fever Viruses Detected in Wild Boar in Hong Kong, 2021–2023
Source: Viruses. 2025 Jun 25;17(7):896. doi: 10.3390/v17070896 (PMC12300889; doi:10.3390/v17070896)
Supplement: Supplementary file 1 [file viruses-17-00896-s001.zip › Supplementary figures and table.pdf]

Supplementary Table 1. Comparison of average nucleotide identity (ANI) among the four ASFV strains identified from wild boar, the strain HK202103 (OK358852.1) previously identified in a domestic pig on a farm in the Hong Kong SAR, and the reference strain Georgia 2007/1.

	Georgia 2007/1 (FR682468.2)	ASFV/HKWB2022TP-00522	ASFV/HKWB2021SSW-12112	ASFV/HKWB2022SK-13869	ASFV/HKWB2022S-10414	HK202103 (OK358852.1)
Georgia 2007/1 (FR682468.2)	100	99.9924	99.99	99.985	99.9921	99.977
ASFV/HKWB2022TP-00522	99.9928	100	99.9902	99.9857	99.9912	99.9763
ASFV/HKWB2021SSW-12112	99.9894	99.99	100	99.9847	99.9893	99.9728
ASFV/HKWB2022SK-13869	99.9849	99.9857	99.9846	100	99.9868	99.9697
ASFV/HKWB2022S-10414	99.9815	99.98	99.9787	99.9768	100	99.9659
HK202103 (OK358852.1)	99.9774	99.9769	99.9733	99.9698	99.9773	100

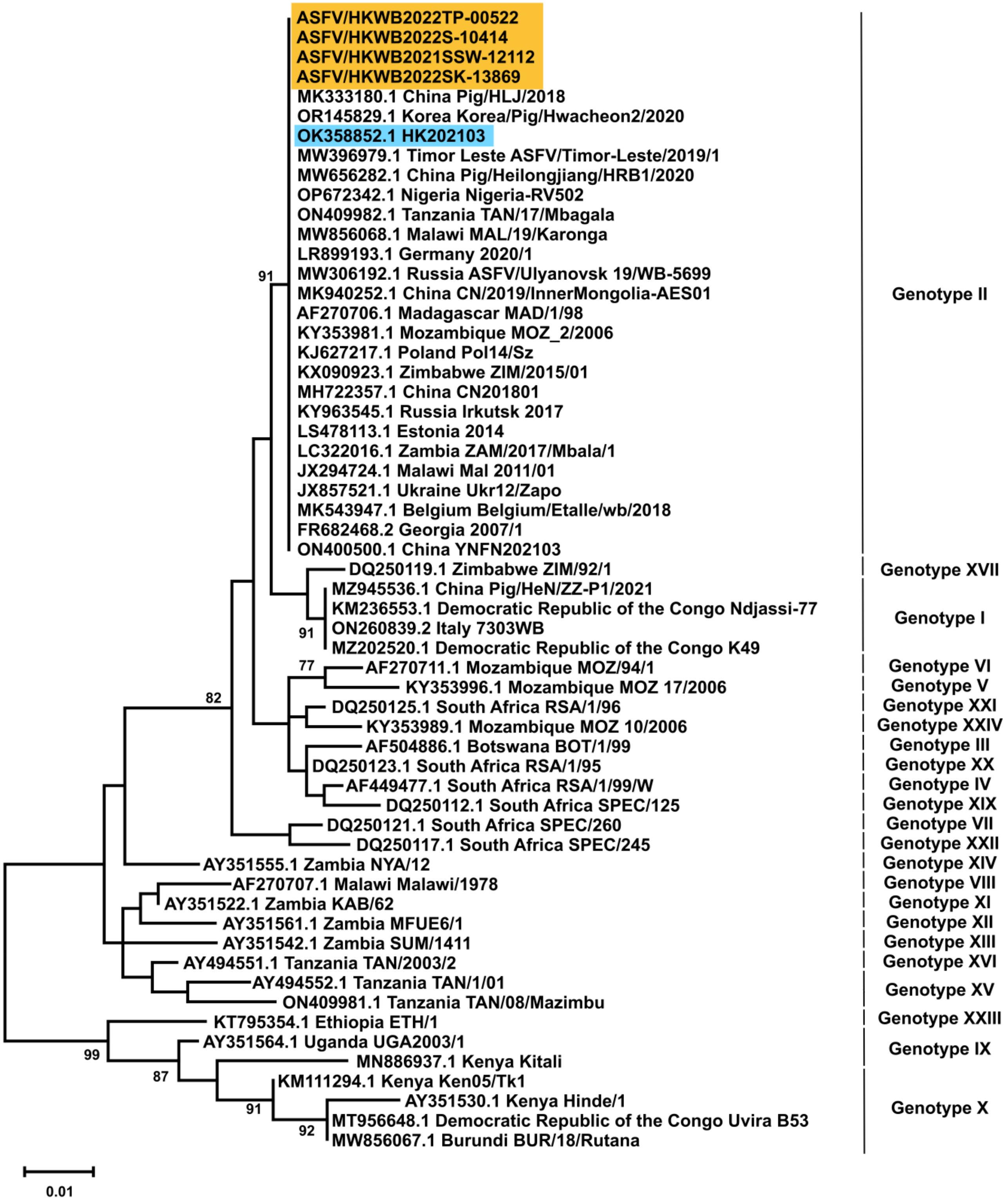

Supplementary Figure 1. P72 genotype tree. A total of 54 sequences of the ASFV B646L partial coding sequence (CDS) from various genotypes, along with the four ASFV strains identified from wild boar (highlighted in orange), and the previously identified ASFV in a domestic pig farm in the Hong Kong SAR (highlighted in blue) are included in the phylogenetic analysis. Only bootstrap values greater than 70 are displayed.

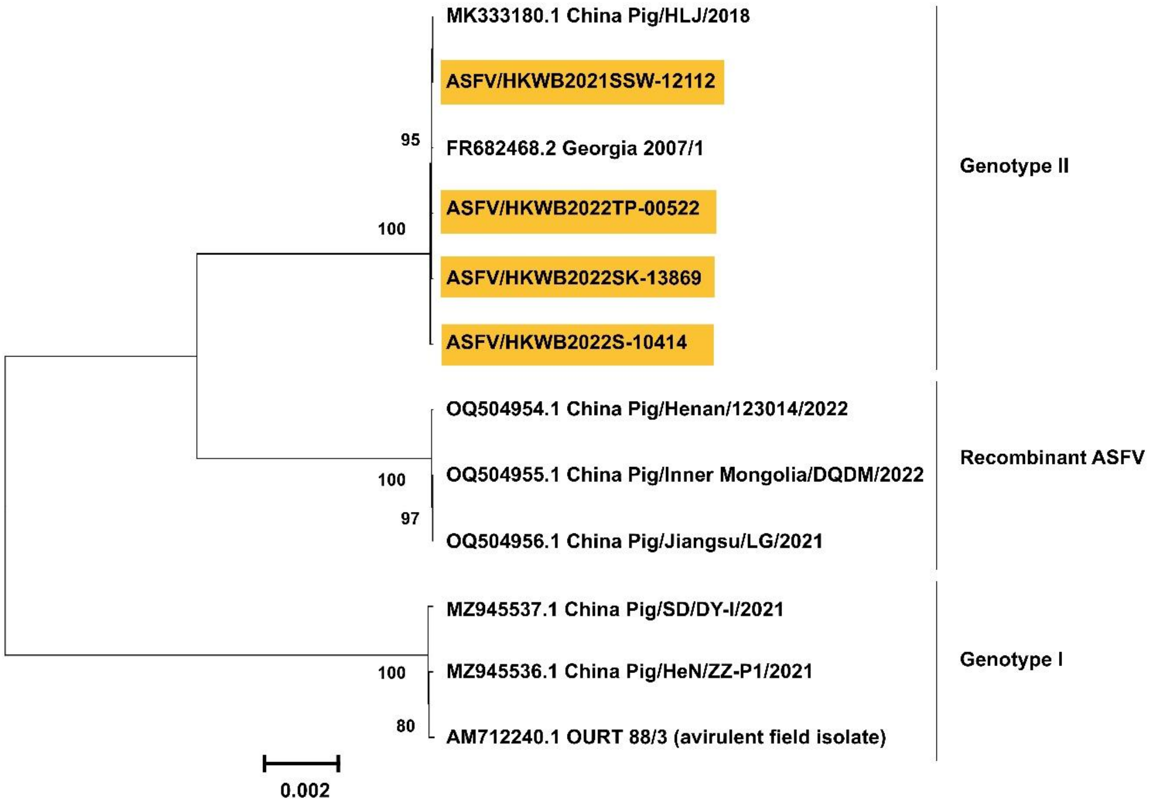

Supplementary Figure 2. UPGMA tree. Four ASFV strains identified from wild boar in this study (highlighted in orange), three genotype I ASFV strains, two ASFV genotype II strains, and three recombinant ASFV strains are included in the phylogenetic analysis. Only bootstrap values greater than 70 are displayed.
