# Supplementary figures and images for: The First African Swine Fever Viruses Detected in Wild Boar in Hong Kong, 2021–2023
Source: Viruses. 2025 Jun 25;17(7):896. doi: 10.3390/v17070896 (PMC12300889; doi:10.3390/v17070896)

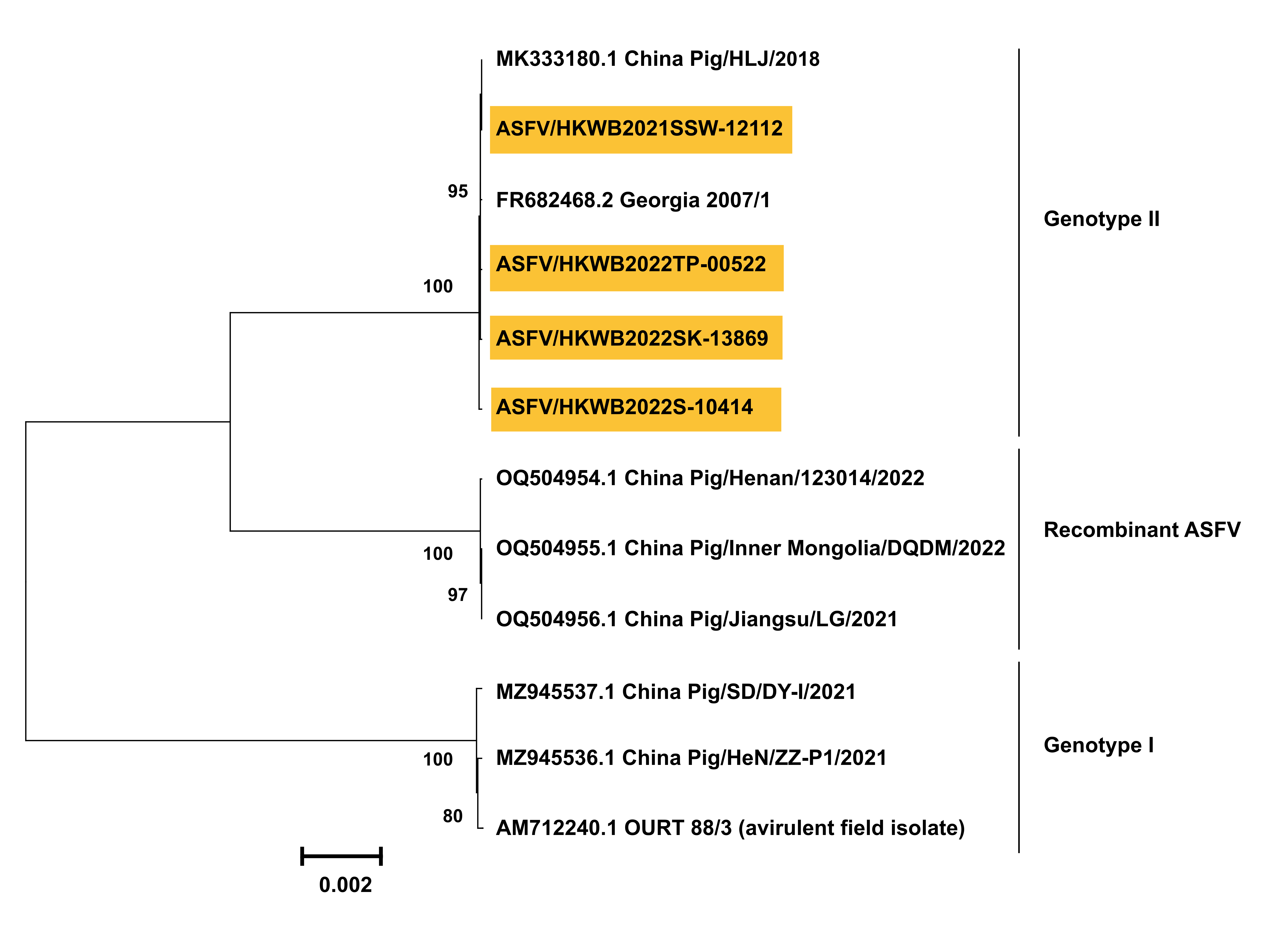

Supplement: Supplementary file 1 [file viruses-17-00896-s001.zip › Supplementary_Figure_2_UPGMA_tree.jpg]
